# Overview of sesquiterpenes and chromones of agarwood originating from four main species of the genus *Aquilaria*

**DOI:** 10.1039/c8ra09409h

**Published:** 2019-01-30

**Authors:** Mei Gao, Xiaomin Han, Ying Sun, Hongjiang Chen, Yun Yang, Yangyang Liu, Hui Meng, Zhihui Gao, Yanhong Xu, Zheng Zhang, Jianping Han

**Affiliations:** National Engineering Laboratory for Breeding of Endangered Medicinal Materials, Institute of Medicinal Plant Development, Chinese Academy of Medical Sciences, Peking Union Medical College Malianwabei Road Beijing 100193 P. R. China zhangzheng@implad.ac.cn jphan@implad.ac.cn +86-10-57833363; Tianjin University of Commerce No. 409 Guangrong Road, Beichen District Tianjin 300134 P. R. China; Zhejiang Pharmaceutical College Ningbo 315100 P. R. China; Hainan Provincial Key Laboratory of Resources Conservation and Development of Southern Medicine, Hainan Branch, Institute of Medicinal Plant Development, Chinese Academy of Medical Sciences, Peking Union Medical College Wanning 571533 P. R. China

## Abstract

The main chemical constituents of agarwood are sesquiterpenes and chromones, which can be divided into different categories depending on their molecular skeletons. Agarwoods are obtained from different plant species: *Aquilaria sinensis*, *A. malaccensis*, *A. crassna*, and *A. subintegra*. In this review, we systematically summarized the structures of 367 compounds isolated from agarwoods originating from four main species. We structurally classified all the components into 11 different types and summarized the number of compounds in each type. Different and identical components were obtained by enumerating the chemical compositions of the different species. Knowledge regarding the chemical constituents of agarwoods of different species will aid understanding of the chemical compositions of agarwoods and will subsequently identify similar compounds that can serve as standards for quality control to provide a reference for future studies on agarwoods from different species and to increase their usefulness.

## Abstract 2

The main compounds in agarwood (sesquiterpenes and chromones) are classified and summarized based on their molecular skeletons and plant species.

## Graphical abstract

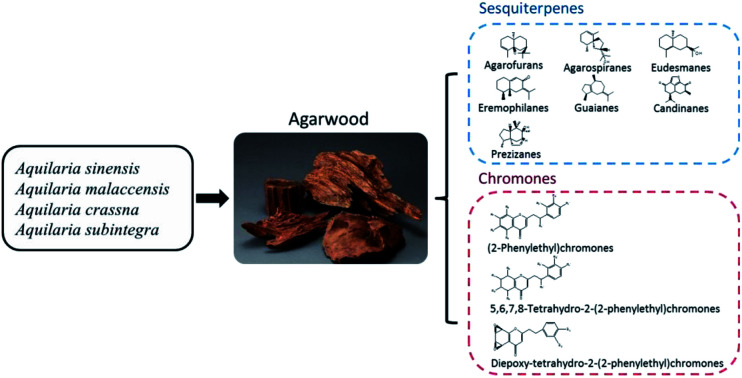

## 1. Introduction

Agarwood is a resinous portion of *Aquilaria* trees, a genus belonging to the family Thymelaeaceae*.* Agarwoods have wide uses in traditional medicine, for example, as aphrodisiacs, sedatives, cardiotonics, and carminatives, as well as in the relief of gastric problems, coughs, rheumatism, and high fever.^1^ In addition, agarwoods are present in important spices and are also used as incense. Agarwood is known as ‘chenxiang’ in Chinese and ‘aloeswood’, ‘agalloch’, ‘eaglewood’, ‘jinkoh’, ‘gaharu’, and ‘kanankoh’ in other parts of the world.^2^

Approximately 15 species of *Aquilaria* are well known for their production of fragrant heartwood, also known as gaharu, aloeswood or agarwood. Wounding of the tree appears to be essential for the initiation of gaharu production, and fungal infection is likely to enhance the process. According to Eurlings *et al.*,^3^ the following nine *Aquilaria* species produce gaharu: *A. beccariana*, *A. crassna*, *A. filaria* (Oken), *A. hirta*, *A. khasiana*, *A. malaccensis*, *A. microcarpa*, *A. rostrata* and *A. sinensis*; these are mainly sourced from India, Southeast Asia, Papua New Guinea, and China (chiefly in Hainan and Guangdong).^3^

As stated in reports, sesquiterpenoids and phenylethyl chromone derivatives are the principal compounds in the oleoresin of agarwood, which are mostly found in the species *Aquilaria*, *A. malaccensis*, *A. agallocha*, *A. sinensis*, and *A. crassna*.^1,4^ In 2016, a review of agarwood noted *A. agallocha* Roxb. (endemic in India), of which the species name is unresolved (Table 1).^5^ The index of CITES species,^6^ Missouri Botanical Garden website,^7^ and the Ayurvedic and Unani Pharmacopoeias all list *A. agallocha* Roxb. as a synonym of *A. malaccensis* Lam. Since then, there have been other reports on this species.^8–10^ Therefore, this article will incorporate all the chemical constituents reviewed in *A. agallocha* into *A. malaccensis*. Other genera of the family Thymelaeaceae, such as *Aetoxylon*, *Gyrinops*, *Phaleria*, and *Gonystylus*, have also been reported to produce agarwoods.^3^ It has been reported that different countries have endemic species; for example, *A. crassna* principally grows in Indochina, *A. malaccensis* is an Indomalesian type found in Malaysia, Thailand and India, and *A. sinensis* is endemic in China. *A. subintegra* is principally found in Thailand.^8^

**Table 1:** Species in the genus *Aquilaria* (accepted names) (The Plant List, 2013)

Species	Authorship
*A. apiculata*	Merr., 1922
*A. baillonii*	Pierre ex Lecomte, 1915
*A. banaense*	P. H. Hô, 1986
*A. banaensis*	P. H. Hô, 1986
*A. beccariana*	Tiegh., 1893
*A. brachyantha*	(Merr.) Hallier L, 1922
*A. citrinicarpa*	(Elmer) Hallier L, 1922
*A. crassna*	Pierre ex Lecomte, 1915
*A. cumingiana*	(Decne.) Ridl., 1901
*A. decemcostata*	Hallier L, 1922
*A. filaria*	(Oken) Merr., 1950
*A. hirta*	Ridl., 1901
*A. khasiana*	Hallier L, 1922
*A. malaccensis*	Lam., 1783
*A. microcarpa*	Baill., 1875
*A. parvifolia*	(Quisumb) Ding Hou, 1960
*A. rostrata*	Ridl., 1924
*A. rugosa*	K. Le-Cong and Kessler, 2005
*A. sinensis*	(Lour.) Spreng., 1825
*A. subintegra*	Ding Hou, 1964
*A. urdanetensis*	(Elmer) Hallier L, 1922
*A. yunnanensis*	S. C. Huang, 1985

All nine of the known *Aquilaria* species can produce agarwood; however, it is not known which species are most productive. Studies have shown that specific species produce specific chemical components which are quite different from one another.^11^ Assessing the similarities and differences between these components is a good way to identify species, determine their quality and classify specific species.

Knowledge of the main constituents of agarwoods and their major differences between species would be a tremendous help in identifying the different species of agarwood and improving their quality and efficacy of use in traditional medicine. This review will focus on species that are frequently used and reported, including *A. malaccensis* (synonymous with *A. agallocha*), *A. sinensis*, and *A. crassna*, and will describe the main chemical constituents of agarwoods from different species. The reference content of this article mainly includes literature abstracts and full-text articles from journals, books, reports and electronic searches, including Google Scholar, Elsevier, PubMed, Springer, Web of Science and other related websites. We have selected nearly one hundred articles from different countries, which have been investigated, analyzed and included in this review. This review discusses compounds that have been isolated since 1963.

## 2. Sesquiterpenes of agarwoods

**Scheme 1:**
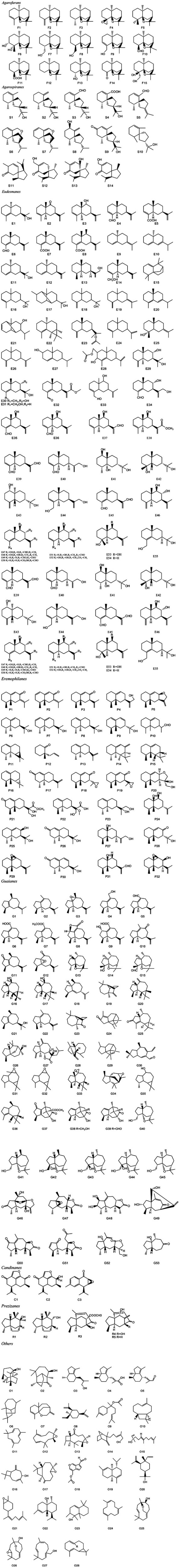
Different types of sesquiterpenes in agarwood.

Agarwoods contain various types of sesquiterpenes, which can be divided into several categories depending on their molecular skeletons. Some examples of these sesquiterpenes are agarofurans, agarospiranes, eudesmanes, eremophilanes, guaianes, candinanes, and prezizanes (shown in Scheme 1). Other compounds are also present in small amounts (shown in Scheme 1).

Almost all types of sesquiterpenes can be found in the following four agarwood species: *A. sinensis*, *A. malaccensis*, *A. crassna*, and *A. subintegra*; however, there are significant differences among the sesquiterpenes of these species, as described in Table 2.

**Table 2:** Sesquiterpenes from different species^a^^,^^b^^,^^c^

No.	Compounds and names	Species			
		*A. s*	*A. m*	*A. c*	*A. su*
	***Agarofurans***				
F1	α-Agarofuran	12 and 13	14 and 15	16	—
F2	β-Agarofuran	12, 13 and 17	15 and 18	16	16
F3	Dihydro-β-agarofuran	13	15 and 16	16	16
F4	Epoxy-β-agarofuran	—	19	—	—
F5	4-Hydroxy-dihydro-agarofuran	13	20	—	—
F6	3,4-Dihydroxydihydroagarofuran	—	20	—	—
F7	Baimuxinol	13 and 21	—	—	—
F8	Isobaimuxinol	12 and 13	—	—	—
F9	Dehydrobaimuxinol	21	—	—	—
F10	(1*S*,2*S*,6*S*,9*R*)-6,10,10-Trimethyl-11-oxatricyclo[7.2.1.01,6]dodecane-2-carbaldehyde	—	19	—	—
F11	Baimuxifuranic acid	13, 22 and 23	—	—	—
F12	(1*R*,6*S*,9*R*)-6,10,10-Trimethyl-11-oxatricyclo[7.2.1.01,6]dodecane	—	24	—	—
F13	(1*R*,2*R*,6*S*,9*R*)-6,10,10-Trimethyl-11-oxatricyclo[7.2.1.01,6]dodecan-2-ol	—	24	—	—
F14	Nor-keto-agarofuran	—	20 and 25	16	16
F15	4-Hydroxyl-baimuxinol	26	—	—	—
	***Agarospiranes***				
S1	(2*R*,5*R*,10*R*)-*a*,*a*,6,10-tetramethyl-spiro[4,5]dec-6-ene-2-methanol (agarospirol)	13, 27 and 28*	16 and 29–31	16	16
S2	Isoagarospirol	—	18	—	—
S3	Oxo-agarospirol (baimuxinal)	13, 17, 23, 27, 28* and 32	14, 18, 33 and 34	16	16
S4	Baimuxinic acid (Bai Mu Xiang acid)	17 and 27	—	—	—
S5	*rel*-(5*R*,10*R*)-2-Isopropylidene-10-methyl-spiro[4.5]dec-6-ene-6-carbaldehyde(vetispira-2(11),6-dien-14-al)	—	25	—	—
S6	*rel*-(1*R*,2*R*)-9-Isopropyl-2-methyl-8-oxatricyclo[7.2.1.01,6]dodec-5-ene(2,14-epoxy-vetispir-6-ene)	—	25	—	—
S7	*rel*-(1*R*,2*R*)-9-Isopropyl-2-methyl-8-oxatricyclo[7.2.1.01,6]dodeca-4,6-dien(2,14-epoxy-vetispira-6(14),7-diene)	—	25	—	—
S8	*rel*-(5*R*,7*S*,10*R*)-2-Isopropylidene-10-methyl-6-methylene-spiro[4.5]decan-7-ol(vetispira-2(11),6(14)-dien-7-ol)	—	25	—	—
S9	(4*R*,5*R*,7*R*)-1(10)-Spirovetiven-11-ol-2-one	23	35	—	—
S10	Hinesol	2, 13 and 36	—	—	—
S11	Acorenone B	—	16	16	16
S12	4-*epi*-15-Hydroxyacorenone	37 and 38*	—	—	—
S13	4-*epi*-10-Hydroxyacoronene	37	—	—	—
S14	15-Hydroxyacorenone	23			
	***Eudesmanes***				
E1	10-*epi*-γ-Eudesmol	13	14, 18 and 25	16	16
E2	(5*S*,7*S*,10*S*)-(−)-Selina-3,11-dien-9-one	—	16 and 34	16	16
E3	(5*S*,7*S*,9*S*,10*S*)-(+)-Selina-3,11-dien-9-ol	—	16 and 34	16	16
E4	Selina-3,11-dien-14-al		34 and 39	16	16
E5	Selina-3,11-dien-14-oic acid (as methyl ester)	—	39	—	—
E6	Selina-4,11-dien-14-al	—	16 and 39	16	16
E7	Selina-4,11-dien-14-oic acid (as methyl ester)	—	39	—	—
E8	9-Hydroxy-selina-4,11-dien-14-oic acid (as methylester)	—	39	—	—
E9	(*S*)-4*a*-Methyl-2-(1-methylethylidene)-1,2,3,4,4*a*,5,6,7-octahydronaphthalene	13	24	—	—
E10	(*S*)-4*a*-Methyl-2-(1-methylethyl)-3,4,4*a*,5,6,7-hexahydronaphthalene	13	24	—	—
E11	(2*R*,4*aS*)-2-(4*a*-Methyl-1,2,3,4,4*a*,5,6,7-octahydronaphthyl)-propan-2-ol(4-nor-*epi*-γ-eudesmol)	13	24	—	—
E12	(2*R*,4*aS*)-4*a*-Methyl-2-(1-methylethenyl)-1,2,3,4,4*a*,5,6,7-octahydronaphthalene	—	24	—	—
E13	Agarol (11(13)-eudesmen-12-ol)	—	40 and 41	—	—
E14	Selina-3,11-dien-14-ol	—	—	16	16
E15	Isolongifolene	36	—	—	—
E16	α-Eudesmol	42	—	—	—
E17	α-Copaen-11-ol	2	—	—	—
E18	β-Eudesmol	13 and 42	16	16	16
E19	γ-Selinene	36, 42 and 43	—	—	—
E20	δ-Selinene	36	—	—	—
E21	α-Copaene-8-ol	43	—	—	—
E22	β-Maaliene	36	—	—	—
E23	β-Eudesmol acetate	—	—	16	16
E24	α-Selinene	2	—	44	—
E25	Eudesm-7(11)-en-4*a*-ol	2	—	—	—
E26	Naphthalene, decahydro-7-isopropenyl-4*a*-methyl-1-methylene-	—	45-	—	—
E27	6-Isopropenyl-4,8*a*-dimethyl-1,2,3,5,6,7,8,8*a*-octahydro-naphthalen-2-ol	2	—	—	—
E28	Acetic acid, 3-hydroxy-6-isopropenyl-4,8*a*-dimethyl-1,2,3,5,6,7,8,8*a*-octahydronaphthalen-2-yl ester	2	—	—	—
E29	5-Desoxylongilobol	23 and 46	—	46	—
E30	Eudesma-4-en-8,11-diol	—	—	47	—
E31	Eudesma-4-en-11,15-diol	23	—	47	—
E32	Methyl-15-oxo-eudesmane-4,11(13)-dien-12-oate	—	—	47	—
E33	Selina-3,11-dien-9,15-diol	48*	—	—	—
E34	(7*S*,8*R*,10*S*)-(+)-8,12-Dihydroxy-selina-4,11-dien-14-al	49*	—	—	—
E35	(7*S*,9*S*,10*S*)-(+)-9-Hydroxy-selina-4,11-dien-14-al	23 and 49*	—	—	—
E36	(5*S*,7*S*,9*S*,10*S*)-(−)-9-Hydroxy-selina-3,11-dien-14-al	49*	—	—	—
E37	(5*S*,7*S*,9*S*,10*S*)-(+)-9-Hydroxy-selina-3,11-dien-12-al	23 and 49*	—	—	—
E38	(5*S*,7*S*,9*S*,10*S*)-(+)-9-Hydroxy-eudesma-3,11(13)-dien-12-methylester	23 and 49*	—	—	—
E39	Selina-3,11-diene-12,15-dial (=12,15-dioxo-α-selinen)	32 and 49*	—	—	—
E40	(4αβ,7β,8αβ)-3,4,4α,5,6,7,8,8α-Octahydro-7-[1-(hydroxymethyl)ethenyl]-4α-methylnaphthalene-1-carboxaldehyde	23 and 49*	50	—	—
E41	Eudesmane-1β,5α,11-triol	49*	—	—	—
E42	(−)-7β*H*-Eudesmane-4α,11-diol	49*	—	—	—
E43	*ent*-4(15)-Eudesmen-11-ol-1-one	49*	—	—	—
E44	15-Hydroxyl-12-oxo-α-selinen	49*	—	—	—
E45	Selina-4,11-diene-12,15-dial	32	50	—	—
E46	(+)-Eudesma-4(14),11(13)-dien-8α,9β-diol	23	—	—	—
E47	(+)-9β-Hydroxyeudesma-4,11(13)-dien-12-al	23	—	—	—
E48	(+)-Eudesma-4,11(13)-dien-8α,9β-diol	23	—	—	—
E49	12,15-Dioxo-selina-4,11-dine	23	—	—	—
E50	12-Hydroxy-4(5),11(13)-eudesmadien-15-al	23	—	—	—
E51	(+)-8α-Hydroxyeudesma-3,11(13)-dien-14-al	23	—	—	—
E52	(+)-Eudesma-3,11(13)-dien-8α,9β-diol	23	—	—	—
E53	(4*R*,5*R*,7*S*,9*S*,10*S*)-(−)-Eudesma-11(13)-en-4,9-diol	23	—	—	—
E54	Selin-11-en-4α-ol	23	—	—	—
E55	Eudesm-4-ene-11,15-diol	23	50	—	—
	***Eremophilanes***				
P1	(+)-(4*S*,5*R*)-Dihydrokaranone	13 and 51	18 and 34	—	—
P2	(+)-(4*S*,5*R*)-karanone	—	18	16	16
P3	Eremophila-9,11-dien-8-one (neopetasane)	2, 13, 26, 38* and 51	16, 33 and 39	16	16
P4	*rel*-(2*R*,8*R*,8*aS*)-2-(1,2,3,5,6,7,8,8*a*-Octahydro-8	—	25	—	—
P5	8,12-Epoxy-eremophila-9,11(13)-diene	28*	25	—	—
P6	(−)-(4*R*,5*S*,7*R*)-Jinkoh-eremol	13	25, 30 and 39	16	16
P7	Dehydro-jinkoh-eremol	—	16, 25 and 39	16	16
P8	(+)-(4*R*,5*S*,7*R*)-Kusunol (=valerianol)	13, 38* and 52	14, 25 and 30	16	16
P9	*rel*-(2*R*,8*S*,8*aS*)-2-(1,2,6,7,8,8*a*-Hexahydro-8,8*a*-dimethyl-2-naphthyl)-propan-2-ol(valenca-1(10),8-dien-11-ol)	—	25	—	—
P10	Valenc- or eremophil-9-en-12-al(tentative)	—	25	—	—
P11	Calarene	—	53	—	—
P12	2,*t*-3-Dimethyl-*r*-2-(3-methyl-2-butenyl)-1-cyclohexanone	—	19	—	—
P13	Valencene	42 and 43	—	54	—
P14	Aristolone	—	—	54	—
P15	Aristolene	42	—	—	—
P16	Nootkatone	42	—	—	—
P17	Calarene	—	—	54	—
P18	7*b-H*-9(10)-ene-11,12-epoxy-8-oxoeremophilane	26	—	—	—
P19	7α-*H*-9(10)-ene-11,12-epoxy-8-oxoeremophilane	26, 38*, 46 and 51	—	46	—
P20	11,13-Dihydroxy-9(10)-ene-8β,12-epoxyemophilane	—	—	46	—
P21	(4*S*,5*R*,7*R*)-11,12-Dihydroxy-eremophila-1(10)-ene-2-oxo-11-methyl ester	—	—	46	—
P22	2-[(2β,8β,8*a*α)-8,8*a*-Dimethyl-1,2,3,4,6,7,8,8*a*-octahydronaphthalen-2-yl]-3-hydroxy-2-methoxpropanoic acid	—	—	47	—
P23	2-[(2β,8α,8*a*α)-8,8*a*-Dimethyl-1,2,3,4,6,7,8,8*a*-octahydronaphthalen-2-yl]propane-1,2-diol	—	—	47	—
P24	(1β,3α,4*a*β,5β,8*a*α)-4,4*a*-Dimethyl-6(prop-1-en-2-yl)octahydronaphtha-lene-1,8*a*(1*H*)-diol	—	—	47	—
P25	(−)-Eremophila-9-en-8β,11-diol	23	—	47	—
P26	11-Hydroxy-valenc-1(10)-en-2-one	23 and 38*	—	—	—
P27	(1β,4αβ,7β,8αβ)-Octahydro-7-[1-(hydroxymethyl)ethenyl]-1,8α-dimethylnaphthalen-4α(2*H*)-ol	23 and 38*	50	—	—
P28	Ligudicin C	51	—	—	—
P29	(+)-9β,10β-Epoxyeremophila-11(13)-en	23	—	—	—
P30	(+)-11-Hydroxyvalenc-1(10),8-dien-2-one	23	—	—	—
P31	2-[(2β,4αβ,8β,8αβ)-Decahydro-4α-hydroxy-8,8α-dimethylnaphthalen-2-yl]prop-2-enal	23	50	—	—
P32	(1αβ,2β,3β,4αβ,5β,8αβ)-Octahydro-4α,5-dimethyl-3-(1-methylethenyl)-3*H*-naphth[1,8*a-b*]oxiren-2-ol	—	50	—	—
	***Guaianes***				
G1	α-Guaiene	—	16 and 34	16	16
G2	α-Bulnesene	—	34	16	16
G3	(−)-Epoxyguai-11-ene (epoxybulnesene)	—	16 and 55	16	16
G4	(−)-Guaia-1(10),11-dien-15-ol	—	16 and 55	16	16
G5	(−)-Guaia-1(10),11-dien-15-al	—	34	—	—
G6	(−)-Guaia-1(10),11-diene-15-carboxylic acid	—	55	—	—
G7	Methyl guaia-1(10),11-diene-15-carboxylate	—	55	—	—
G8	(−)-Guaia-1(10),11-dien-15,2-olide	—	55	—	—
G9	(−)-2α-Hydroxyguaia-1(10),11-dien-15-oic acid (identified in acidic fraction as Me-ester)	—	55 and 56	—	—
G10	(+)-Guaia-1(10),11-dien-9-one	—	55	—	—
G11	Rotundone	—	55	—	—
G12	(+)-1,5-Epoxy-nor-ketoguaiene	—	39	—	—
G13	*epi*-Ligulyl oxide	13	—	—	—
G14	Sinenofuranol	13, 17, 22 and 57	—	—	—
G15	Sinenofuranal	17	—	—	—
G16	Viridiflorol	13 and 42	—	—	—
G17	Ledol	42	—	—	—
G18	γ-Gurjunene	42	—	—	—
G19	Longifolene	58	—	—	—
G20	Aromadendrene	—	—	54	—
G21	Guaiol	36	—	—	—
G22	δ-Guaiene	—	—	44	—
G23	3,3,7-Trimethyltri-cycloundecan-8-one	58	—	—	—
G24	Cyperotundone	—	—	16	16
G25	Cyclocolorenone	—	16	16	16
G26	α-Cedrol	36	—	—	—
G27	11β-Hydroxy-13-isopropyl-dihydrodehydrocostus lactone	59	—	—	—
G28	Jumping	—	—	54	—
G29	α-Patchoulene	28* and 60	—	—	—
G30	Velleral	28 and 42	—	—	—
G31	Isoaromadendrene epoxide	2 and 42	—	—	—
G32	Aromadendrene oxide-(1)	2	—	—	—
G33	Aromadendrene oxide-(2)	2	—	—	—
G34	Diepi-α-cederene epoxide	2	—	—	—
G35	1*H*-Cycloprop[*e*]azulen-4-oldecahydro-1,1,4,7-tetramethyl-,[1*aR*-(1*a*.alpha.,4.beta.,4*a*.beta.,7.alpha., 7*a*.beta., 7*b*.alpha.)]-	—	45	—	—
G36	α-Gurjunene	—	—	54	—
G37	Chamaejasmone E	—	61	—	—
G38	Chamaejasmone D	—	61	—	—
G39	Auranticanol A	—	61	—	—
G40	Qinanol A	57	—	—	—
G41	Qinanol B	57	—	—	—
G42	Qinanol C	57	—	—	—
G43	Qinanol D	57	—	—	—
G44	Qinanol E	57	—	—	—
G45	Sinenofuranol	57	—	—	—
G46	3-Oxo-7-hydroxylholosericin A	38*	—	—	—
G47	1,5;8,12-Diepoxyguaia-12-one	38*	—	—	—
G48	Qinanlactone	37	—	—	—
G49	Qinan-guaiane-one	37	—	—	—
G50	7*H*-Guaia-1(10)-en-12,8-olide	32	—	—	—
G51	1,10-Dioxo-4α*H*-5α*H*-7β*H*-11α*H*-1,10-secoguaia-2(3)-en-12,8β-olide	32	—	—	—
G52	1α-Hydroxy-4β*H*-5β*H*-7β*H*-11α*H*-8,9-secoguaia-9(10)-en-8,12-olide	32	—	—	—
G53	1α-Hydroxy-4α,10α-dimethyl-5βH-octahydro-azulen-8-one	32	—	—	—
	***Candinanes***				
C1	8β*H*-Dihydrogmelofuran	—	62	—	—
C2	Gmelofuran	—	62	—	—
C3	(7β,8β,9β)-8,9-Epoxycalamenen-10-one	—	—	46	—
	***Prezizanes***				
R1	Jinkohol	—	30 and 31	—	—
R2	Jinkohol II	—	30	—	—
R3	Daphnauranol B	—	61	—	—
R4	Daphnauranol C	—	61	—	—
R5	Daphnauranol D	—	61	—	—
	***Others***				
O1	Patchoulialcohol	43	—	—	—
O2	(+)-8β-Hydroxy-longicamphenylone	59	—	—	—
O3	Valerenol	—	—	54	—
O4	Valerenic acid	42	—	54	—
O5	Valerenal	28*	—	54	—
O6	Dihydro-neoclovene	—	—	54	—
O7	2,6-Dimethyl-10-methylene-12-oxatricyclo[7.3.1.0(1,6)]tridec-2-ene	2	—	—	—
O8	β-Elemene	—	—	16	16
O9	α-Bisabolol acetate	—	—	—	16
O10	β-Caryophyllene	43	—	—	—
O11	α-Humulene	43	—	—	—
O12	Humulene diepoxide A	58	—	—	—
O13	Kobusone	58	—	—	—
O14	Santalol	36, 42 and 58	—	—	—
O15	(*E*)-Nerolidol	—	16 and 18	16	16
O16	Caryophyllenol-II	58	—	—	—
O17	Caryophylleneoxide	2, 42 and 43	45	—	—
O18	Baldrinal	28*	—	—	—
O19	α-Muurolene	28* and 63	—	—	—
O20	Elemol	2 and 13	—	—	—
O21	*cis-Z*-α-Bisabolene epoxide	2	—	—	—
O22	Cubenol	2	—	—	—
O23	1,2,5,5,8*a*-Pentamethyl-1,2,3,5,6,7,8,8*a*-octahydronaphthalen-1-ol	2	—	—	—
O24	1,5,9-Trimethyl-1,5,9-cyclododecatriene	38*	—	—	—
O25	Aquilanol A	—	61	—	—
O26	Aquilanol B	—	61	—	—
O27	12-Hydroxyhumula-2*Z*,6*E*,9*E*-triene	—	61	—	—
O28	14-Hydroxy-α-humulene	23	—	—	—

a *A. s*, *A. m*, *A. c*, and *A. su* indicate *A. sinensis*, *A. malaccensis*, *A. crassna*, and *A. subintegra*, respectively.

b The reference was not found.

c “*” indicates that the agarwood in this article was artificial agarwood.

### 2.1. Sesquiterpenes in *A. sinensis*

The sesquiterpenoids of agarwood are mainly derived from agarwood oil. Early publications on agarwood essential oils reflect the fact that the agarwood resin components are separated by solvent extraction, followed by column chromatography for purification and structural analysis using spectroscopy, including NMR. For example, Yang *et al.*^12,21,27^ and Xu *et al.*^17^ isolated sesquiterpenes F1–F2, F7–F9, F11, S1–S3, and G14 from *A. sinensis*. Yang and coworkers^64^ isolated G19, G23, O12–O13, and O16 from ethanol and petrol ether extracts of *A. sinensis* and later found two new sesquiterpenes, G27 and O2.

Later articles focused on the use of “combination” techniques to detect and identify compounds. For example, Mei *et al.*,^13,42^ Tian *et al.*,^36^ Deng *et al.*,^43^ Chen *et al.*,^2^ and Miao *et al.*^63^ detected F3, F5, S10, E1, E9–E11, E15–E22, E24–E28, P1, P3, P6, P8, P13, P15–P16, G13–G14, G16–G18, G21, G26, G30–G34, O1, O5, O7, O10–O11, O17, O20, and O19–O23 from essential oils of *A. sinensis* by GC/MS. Lin *et al.* investigated agarwood obtained from fungus-infected *Aquilaria* at different times by GC-MS and showed the presence of S1, S3, P5, G29–G30, O5, and O18–O19.^28^ GC-MS combined with multivariate data analysis was used to construct chemical profiles of natural and artificial agarwoods. The chemical composition of agarwood oil was also studied. Agarwood essential oils are produced by steam distillation or the latest supercritical fluid extraction techniques.

With the development of separation technology, increasing numbers of publications are reporting the separation of sesquiterpenoids from extracts of agarwood resin. The purpose of these studies is to isolate and purify compounds from agarwood, to explore the pharmacological activities of these compounds, and to guide the selection of quality indicators and clinical medication. ‘Qi-Nan’ is regarded to have the highest quality and is therefore the most expensive agarwood in the market; Yang and coworkers^26,37,57^ performed studies on ‘Qi-Nan’ originating from *A. sinensis* and characterized some new sesquiterpenes, including F15, P18, P19, P27, G40–G44, G14, G48, G49, S13, and S12, from the Et_2_O extract of agarwood. From the ethanolic (EtOH) extract of agarwood induced by artificial holing, Li *et al.*^38,49^ isolated and identified two new guaiane-type sesquiterpenoids (G46, G47) and eleven eudesmane-type sesquiterpenoids (E34–E44) together with some known sesquiterpenoids, S12, O24, P3, P8, P19, P26, and P27. Kuang and coworkers^48^ were also interested in agarwood induced by artificial holing; they researched the chemical constituents of the *n*-butanol fraction of an EtOH extract and thereby obtained one new sesquiterpene, E33. Huo *et al.*^23^ obtained nine new sesquiterpenes together with seventeen known ones (E29, E31, E35, E37, E38, E40, E47–E54, F11, P24–P27, P29, P30, O28, S3, S9, and S14) from a 95% EtOH extract of resinous wood. Zhao *et al.*^32^ isolated sesquiterpenoids G50–G53, S3, E39, and E45 from the 95% EtOH extract of eaglewood of *A. sinensis*. Additionally, four sesquiterpenes, E31, P1, P19, and P28, were isolated from the resinous wood of *A. sinensis* in 2018.^51^

### 2.2. Sesquiterpenes in *A. malaccensis* (or *A. agallocha*)

*A. malaccensis* is found in Malaysia, Thailand and India and is currently the most widely distributed species.^8^ The researchers who study this variety are also widely geographically distributed, including Japan, Switzerland, and China. In 1963, Maheshwari and Jain^15,20,41^ isolated and identified F1–F3, F6–F7, F14, and E13 from *A. agallocha*. From 1992 to 1995, Näf and coworkers found 21 new sesquiterpenes, including agarofurans (F4, F10, and F12–F14), agarospiranes (S5–S8), five eudesmanes (E1, E9–E12), and seven eremophilanes (P4–P10 and P12).^19,24,25^ Ishihara, T. Tsuneya and coworkers found seven eudesmane-type sesquiterpenes (E2–E8) and 12 guaiane-type sesquiterpenes (G1–G12, S3, P1, and P3).^34,39,55^ The first agarospirane-type sesquiterpene, S1,^29,45^ was found in 1964; later, S9 was obtained from the 70% ethanol extract of Vietnamese agarwood.^35^ Later, two candinane-type sesquiterpenes, C1 and C2,^62^ were found. In 2009, Bhuiyan extracted E26 and G35 (among others) from naturally formed agarwood of *A. agallocha* as well as from agarwood formed through manual stimulation.^45^ From 1980 to 1983, Nakanishi^30,31^ discovered three new sesquiterpene alcohols from an agarwood (most likely *A. malaccensis*), named jinkoh-eremol (P6), jinkohol (R1) and jinkohol II(R2), together with S1 and P8, the major sesquiterpene constituents; their structures have all been established. Wu and coworkers^33,50^ discovered four new sesquiterpenoids, P27, P31, P32, and E40, together with the four known sesquiterpenoids E55, E45, P3, and S3, all of which were isolated from a 70% MeOH extract of *A. malaccensis* agarwood chips. Ma *et al.*^61^ reported sesquiterpenoids O25–O27, G37–G39, and R3–R5 from the ether extract of agarwood of *A. malaccensis.*

### 2.3. Sesquiterpenes in *A. crassna* and *A. subintegra*

There are currently few studies on *A. crassna* and *A. subintegra*.^8^ In 2001, Pripdeevech and coworkers analyzed the diversity of compounds in *A. malaccensis*, *A. crassna*, and *A. subintegra* by GC-MS and GC-O. The study suggested that these species share sesquiterpenes F3, F15, S3, S11, E2, E3, E6, E18, P3, P7, G1, G3, G4, G25, O14, and O15.^16^ E24 and G22 were found in the supercritical carbon dioxide extraction of *A. crassna*.^44^ Pornpunyapat, Chetpattananondh and Tongurai, assessed the extraction conditions of essential oils obtained from *A. crassna* and detected P13–P14, P17, G20, G28, G36, and O3–O6.^54^

Wang *et al.*^46^ isolated and identified the sesquiterpenoids C3, P19, E2 and E29 from the ethyl acetate (EtOAc) extract of agarwood originating from *A. crassna*. The following year, Kang and Dai *et al.*^47^ separated the sesquiterpenes P22–P25 and E30–E32 from a 95% EtOH extract of agarwood from *A. crassna*.

## 3. Chromone derivatives in agarwoods

**Scheme 2:**
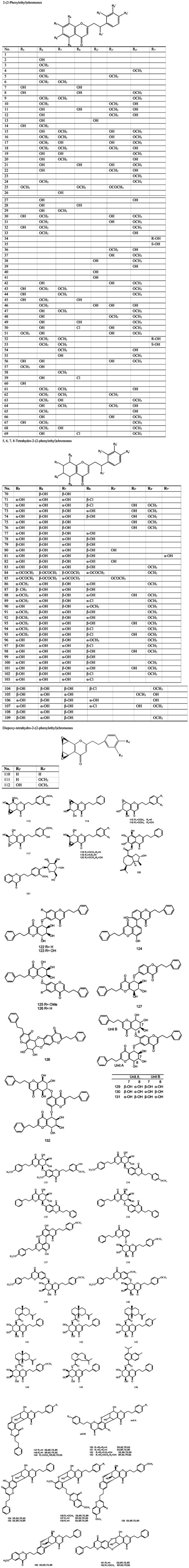
Different types of chromone derivatives in agarwood.

Chromone derivatives are other major constituents of agarwoods. They have been obtained from only a few plant species, including *Eremophila georgei*, *Bothriochloa ischaemum* (Gramineae), and agarwoods originating from *Aquilaria* spp. (Thymelaeaceae). 2-(2-Phenylethyl)chromone derivatives are the characteristic components of agarwoods, and more than 40 such derivatives have been found in agarwoods belonging to different species. Depending on the molecular skeleton, chromones can be divided into 2-(2-phenylethyl)chromones, 5,6,7,8-tetrahydro-2-(2-phenylethyl)chromones, diepoxy-tetrahydro-2-(2-phenylethyl)chromones, and associated chromones (shown in Scheme 2). As shown in Table 3, the variation of chromones in different species is striking.

**Table 3:** Chromones from different species^a^^,^^b^^,^^c^

No.	Compounds and names	Species		
		*A. s*	*A. m*	*A. c*
	**2-(2-Phenylethyl)chromones**			
1	2-(2-Phenylethyl)chromone (flindersiachromone)	51, 65, 76, 84 and 102	33, 63 and 99	—
2	6-Hydroxy-2-(2-phenylethyl)chromone (AH_3_)	65, 76, 79* and 102	33 and 86	103
3	6-Methoxy-2-(2-phenylethyl)chromone (AH_4_)	65 and 102	33 and 86	—
4	6-Hydroxy-2-[2-(4-methoxyphenyl)ethyl]chromone	65, 76 and 79*	—	—
5	6-Methoxy-2-[2-(3-methoxyphenyl)ethyl]chromone (AH_5_)	65 and 79*	50 and 86	—
6	6,7-Dimethoxy-2-(2-phenylethyl)chromone (AH_6_)	51, 65, 79* and 102	33 and 86	—
7	5,8-Dihydroxy-2-(2-phenylethyl)chromone (AH_7_)	66 and 84	91	—
8	5,8-Dihydroxy-2-[2-(4-methoxyphenyl)ethyl]chromone	66		—
9	6,7-Dimethoxy-2-[2-(4′-methoxyphenyl)ethyl]chromone (AH_8_)	48*, 51 and 66	33 and 91	—
10	6-Methoxy-2-[2-(3-methoxy-4-hydroxyphenyl)ethyl]chromone	67 and 79*	80 and 99*	—
11	6,8-Dihydroxy-2-[2-(3-methoxy-4-hydroxyphenyl)ethyl]chromone	67	—	—
12	6-Hydroxy-2-[2-(3-methoxy-4-hydroxyphenyl)ethyl]chromone	68 and 80*	—	—
13	6-Hydroxy-2-(2′-hydroxy-2-phenylethyl)chromone	76 and 104	—	—
14	5-Hydroxy-6-methoxy-2-(2-phenylethyl)chromone	104	—	—
15	6-Hydroxy-7-methoxy-2-[2-(3′-hydroxy-4′-methoxyphenyl)ethyl]chromone	73, 75 and 80*	—	—
16	6,7-Dimethoxy-2-[2-(3′-hydroxy-4′-methoxyphenyl)ethyl]chromone	73 and 80*	—	—
17	7-Hydroxy-6-methoxy-2-[2-(3′-hydroxy-4′-methoxy-phenyl)ethyl]chromone	73	—	—
18	6,7-Dimethoxy-2-[2-(4′-hydroxy-3′-methoxyphenyl)ethyl]chromone	73	—	—
19	6,7-Dihydroxy-2-[2-(4′-methoxyphenyl)ethyl]chromone	73 and 80*	—	—
20	6-Hydroxy-7-methoxy-2-[2-(4′-hydroxyphenyl)ethyl]chromone	73	—	—
21	6,8-Dihydroxy-2-[2-(3′-hydroxy-4′-methoxyphenyl)ethyl]chromone	73 and 75	—	—
22	6-Hydroxy-2-[2-(4′-hydroxy-3′-methoxyphenyl)ethenyl]chromone	73 and 76	—	—
23	2-[2-(4′-Methoxyphenyl)ethyl]chromone	76, 84 and 105	50 and 90	—
24	6-Methoxy-2-[2-(4′-methoxyphenyl)ethyl]chromone	79*	50 and 90	—
25	7,8-Dimethoxy-2-[2-(3′-acetoxyphenyl)ethyl]chromone	—	98	—
26	7-Hydroxy-2-(2-phenylethyl)chromone	—	99	—
27	6-Hydroxy-2-[2-(4-hydroxyphenyl)ethyl]chromone	—	99	—
28	6,8-Dihydroxy-2-(2-phenylethyl)chromone	79*	99	—
29	6-Hydroxy-7-methoxy-2-(2-phenylethyl)chromone	51	99	—
30	5-Hydroxy-6-methoxy-2-[2-(3-hydroxy-4-methoxyphenyl)ethyl]chromone	79*	—	—
31	6-Methoxy-2-[2-(3-hydroxy-4-methoxyphenyl)ethyl]chromone	79* and 80*	—	—
32	5-Hydroxy-6-methoxy-2-[2-(4-methoxyphenyl)ethyl]chromone	79*	—	—
33	6-Methoxy-2-[2-(4′-hydroxyphenyl)ethyl]chromone	79*	—	—
34	(*R*)-2-(2-Hydroxy-2-phenylethyl)chromone	—	—	103
35	(*S*)-2-(2-Hydroxy-2-phenylethyl)chromone	—	—	103
36	2-[2-(3-Methoxy-4-hydroxyphenyl)ethyl]chromone (qinanones B)	76	—	103
37	2-[2-(3-Hydroxy-4-methoxyphenyl)ethyl]chromone (qinanones A)	76	—	—
38	2-[2-(2-Hydroxy-4-methoxyphenyl)ethyl]chromone (qinanones C)	76	—	103
39	2-[2-(4-Hydroxyphenyl)ethyl]chromone (qinanones D)	76	—	—
40	2-[2-(3-Hydroxyphenyl)ethyl]chromone (qinanones E)	76	—	—
41	Qinanone F	76	—	—
42	6-Hydroxy-2-[2-(3-hydroxy-4-methoxyphenyl)ethyl]chromone	76 and 80*	—	—
43	5-Hydroxy-6,7-dimethoxy-2-[2-(4′-methoxyphenyl)ethyl]chromone	84	—	—
44	5-Hydroxyl-7-methoxy-2-[2-(4′-methoxyphenyl)ethyl]chromone	83*	—	—
45	5,8-Dihydroxy-6-methoxy-2-(2-phenylethyl)chromone	83*	—	—
46	6-Methoxy-2-[2-(2′,3′,4′-trihydroxy)phenylethyl]chromone	83*	—	—
47	6-Hydroxy-7-methoxy-2-[2-(4-methoxyphenyl)ethyl]chromone	80*	—	—
48	6-Hydroxy-2-[2-(3,4-dimethoxyphenyl)ethyl]chromone	80*	—	—
49	6,8-Dihydroxy-2-[2-(4-methoxyphenyl)ethyl]chromone	80*	—	—
50	8-Chloro-6-hydroxy-2-[2-(3-methoxy-4-hydroxyphenyl)ethyl]chromone	80*	—	—
51	5-Methoxy-6-hydroxy-2-[2-(3-methoxy-4-hydroxyphenyl)ethyl]chromone	80*	—	—
52	(*R*)-6,7-Dimethoxy-2-(2-hydroxy-2-phenylethyl)chromone	80*	—	—
53	(*S*)-6,7-Dimethoxy-2-(2-hydroxy-2-phenylethyl)chromone	80*	—	—
54	7-Methoxy-2-[2-(4′-hydroxy-phenyl)ethyl]chromone	51	—	—
55	7-Hydroxy-2-[2-(4′-methoxyphenyl)ethyl]chromone	51	—	—
56	5,6-Dihydroxy-2-[2-(3′-hydroxy-4′-methoxyphenyl)ethyl]chromone	51	—	—
57	6-Hydroxy-5-methoxy-2-(2-phenyl-ethyl)chromone	51	—	—
58	7-Methoxy-2-(2-phenylethyl)chromone	51 and 84	50	—
59	8-Chloro-6-hydroxy-2-(2-phenylethyl)chromone	74 and 84	—	—
60	5-Hydroxy-2-(2-phenylethyl)chromone	84	—	—
61	6,7-Dimethoxy-2-[2-(4-hydroxyphenyl)ethyl]chromone	79* and 80*	—	—
62	6,7-Dimethoxy-2-[2-(3-methoxy-4-hydroxyphenyl)ethyl]chromone	80*	—	—
63	6-Methoxy-7-hydroxy-2-[2-(4-methoxyphenyl)ethyl]chromone	80*	—	—
64	4′,6-Dihydroxy-3′,7-dimethoxy-2-(2-phenyl)ethylchromone (aquilarone G)	75	—	—
65	4′-Hydroxy-6-methoxy-2-(2-phenylethyl)chromone (aquilarone H)	75	—	—
66	3′,6-Dihydroxy-4′-methoxy-2-(2-phenylethyl)chromone (aquilarone I)	75	—	—
67	5-Hydroxy-6-methoxy-2-[2-(4-methoxyphenyl)ethyl]-4*H*-1-benzopyran-4-one	—	50	—
68	7-Hydroxy-6-methoxy-2-[2-(4-methoxyphenyl)ethyl]-4*H*-1-benzopyran-4-one	—	33	—
69	8-Chloro-6-hydroxy-2-[2-(4-methoxyphenyl)ethyl]chromone	74	—	—
	**5,6,7,8-Tetrahydro-2-(2-phenylethyl)chromones**			
70	6,7-Dihydroxy-2-(2-phenylethyl)-5,6,7,8-tetrahydrochromone	104	—	—
71	8-Chloro-2-(2-phenylethyl)-5,6,7-trihydroxy-5,6,7,8-tetrahydrochromone	84, 102 and 104	—	—
72	8-Chloro-5,6,7-trihydroxy-2-(3-hydroxy-4-methoxyphenethyl)-5,6,7,8-tetrahydro-4*H*-chromen-4-one	69	—	—
73	5,6,7,8-Tetrahydroxy-2-(3-hydroxy-4-methoxyphenethyl)-5,6,7,8-tetrahydro-4*H*-chromen-4-one	70	—	—
74	(5*S*,6*R*,7*S*,8*R*)-2-[2-(3′-Hydroxy-4′-methoxyphenyl)ethyl]-5,6,7,8-tetrahydroxy-5,6,7,8-tetrahydrochromone (aquilarone D)	48 and 75	—	—
75	(5*S*,6*R*,7*S*)-5,6,7-Trihydroxy-2-(3-hydroxy-4-methoxyphenethyl)-5,6,7,8-tetrahydro-4*H*-chromen-4-one	71	87	—
76	(5*S*,6*R*,7*R*)-5,6,7-Trihydroxy-2-(3-hydroxy-4-methoxyphenethyl)-5,6,7,8-tetrahydro-4*H*-chromen-4-one	71 and 84	87	—
77	Agarotetrol (AH_1_)	77 and 81*	88	—
78	(5*S*,6*R*,7*S*,8*R*)-2-(2-Phenylethyl)-5*e*′,6*e*,7*e*,8*e*-tetrahydroxy-5,6,7,8-tetrahydrochromone(isoagarotetrol) (AH_2_)	—	88	—
79	(5*R*,6*R*,7*S*,8*R*)-2-(2-Phenylethyl)-5*e*′,6*a*,7*e*,8*e*-tetrahydroxy-5,6,7,8-tetrahydrochromone (AH_16_)	77	94	—
80	5α,6β,7β,8α-Tetrahydroxy-2-[2-(2-hydroxyphenyl)ethyl]-5,6,7,8-tetrahydrochromone (AH_23_)	—	95	—
81	5α,6β,7β-Trihydroxy-8α-methoxy-2-(2-phenylethy)chromone (AH_17_)	81*	95	—
82	5α,6β,7α,8β-Tetrahydroxy-2-[2-(2-hydroxy-phenyl)ethyl]-5,6,7,8-tetrahydrochromone (AH_2b_)	48*	89	—
83	5α,6β,7α,8β-Tetrahydroxy-2-[2-(4-methoxy-phenyl)ethyl]-5,6,7,8-tetrahydrochromone (AH_2a_)	—	89	—
84	5α,6β,7β,8α-Tetraacetoxy-2-[2-(4-methoxy-phenyl)ethyl)]-5,6,7,8-tetrahydrochromone (AH_1A_)	—	89	—
85	(5*S*,6*S*,7*R*)-2-[2-(2-Acetoxyphenyl)ethyl]-5*a*′,6*a*,7*a*-tri-acetoxy-5,6,7,8,8-pentahydrochromone (AH_9_)	—	91	—
86	(5*S*,6*S*,7*R*,8*S*)-2-[2-(4-Methoxyphenyl)ethyl]-6,7,8-trihydroxy-5-methoxy-5,6,7,8-tetrahydrochromone (tetrahydrochromone A)	81*	—	—
87	(5*R*,6*R*,7*S*,8*R*)-2-(2-Phenylethyl)-6,7,8-trihydroxy-5-methoxy-5,6,7,8-tetrahydrochromone (tetrahydrochromone B)	81*	—	—
88	(5*S*,6*S*,7*R*,8*S*)-2-[2-(3′-Hydroxy-4′-methoxyphenyl)ethyl]-6,7,8-trihydroxy-5-methoxy-5,6,7,8-tetrahydrochromone (tetrahydrochromone C)	81*	—	—
89	(5*S*,6*S*,7*R*,8*S*)-2-[2-(4′-Methoxyphenyl)ethyl]-8-chloro-6,7-dihydroxy-5-methoxy-5,6,7,8-tetrahydrochromone (tetrahydrochromone D)	81*	—	—
90	(5*S*,6*R*,7*R*,8*S*)-2-[2-(4′-Methoxyphenyl)ethyl]-5,6,7-trihydroxy-8-methoxy-5,6,7,8-tetrahydrochromone (tetrahydrochromone E)	81*	—	—
91	(5*S*,6*R*,7*S*,8*R*)-2-[2-(4-Methoxyphenyl)ethyl]-6,7,8-trihydroxy-5-methoxy-5,6,7,8-tetrahydrochromone (tetrahydrochromone F)	81*	—	—
92	(5*R*,6*S*,7*R*,8*S*)-2-[2-(4-Methoxyphenyl)ethyl]-6,7,8-trihydroxy-5-methoxy-5,6,7,8-tetrahydrochromone (tetrahydrochromone G)	81*	—	—
93	(5*S*,6*R*,7*S*,8*R*)-2-[2-(3′-Hydroxy-4′-methoxyphenyl)ethyl]-6,7,8-trihydroxy-5-methoxy-5,6,7,8-tetrahydrochromone (tetrahydrochromone H)	81*	—	—
94	(5*S*,6*R*,7*S*,8*R*)-2-[2-(4-Methoxyphenyl)ethyl]-8-chloro-6,7-dihydroxy-5-methoxy-5,6,7,8-tetrahydrochromone (tetrahydrochromone I)	81*	—	—
95	(5*S*,6*R*,7*S*,8*R*)-2-[2-(3′-Hydroxy-4′-methoxyphenyl)ethyl]-8-chloro-6,7-dihydroxy-5-methoxy-5,6,7,8-tetrahydrochromone (tetrahydrochromone J)	81*	—	—
96	(5*S*,6*R*,7*R*,8*S*)-2-[2-(4′-Methoxyphenylethyl)]-5,6,7,8-tetrahydroxy-5,6,7,8-tetrahydrochromone	81*	—	—
97	*rel*-(5*R*,6*S*,7*S*,8*R*)-8-Chloro-5,6,7,8-tetrahydro-5,6,7-trihydroxy-2-[2-(4-methoxyphenyl)ethyl]-4*H*-1-benzopyran-4-one	81*	33	—
98	(5*S*,6*S*,7*S*,8*R*)-2-[2-(3′-Hydroxy-4′-methoxyphenyl)ethyl]-5,6,7,8-tetrahydroxy-5,6,7,8-tetrahydrochromone (aquilarone A)	75 and 81*	—	—
99	(5*S*,6*S*,7*S*,8*R*)-2-(2-Phenylethyl)-5,6,7,8-tetrahydroxy-5,6,7,8-tetrahydrochromone (aquilarone B)	75, 81* and 84	—	—
100	(5*S*,6*S*,7*S*,8*R*)-2-[2-(4′-Methoxyphenyl)ethyl]-5,6,7,8-tetrahydroxy-5,6,7,8-tetrahydrochromone (aquilarone C)	75, 81* and 84	—	—
101	(5*S*,6*R*,7*R*,8*S*)-2-[2-(3′-Hydroxy-4′-methoxyphenyl)ethyl]-5,6,7,8-tetrahydroxy-5,6,7,8-tetrahydrochromone (aquilarone E)	75 and 81*	—	—
102	(5*R*,6*R*,7*R*,8*S*)-8-Chloro-5,6,7-trihydroxy-2-(4-methoxyphenethyl)-5,6,7,8-tetrahydrochromone	84	—	—
103	(5*S*,6*S*,7*S*,8*S*)-8-Chloro-5,6,7-trihydroxy-2-(2-phenylethyl)-5,6,7,8-tetrahydrochromone	84	—	—
104	(5*R*,6*R*,7*R*,8*R*)-8-Chloro-5,6,7-trihydroxy-2-(4-methoxyphenethyl)-5,6,7,8-tetrahydrochromone	84	—	—
105	(5*R*,6*S*,7*S*)-5,6,7-Trihydroxy-2-(4-hydroxy-3-methoxyphenethyl)-5,6,7,8-tetrahydrochromone	84	—	—
106	(5*S*,6*R*,7*R*,8*S*)-2-[2-(4′-Hydroxyphenyl)ethyl]-5,6,7,8-tetrahydroxy-5,6,7,8-tetrahydrochromone (aquilarone F)	75	—	—
107	*rel*-(5*R*,6*S*,7*S*,8*R*)-8-Chloro-5,6,7,8-tetrahydro-5,6,7-trihydroxy-2-[2-(3-hydroxy-4-methoxyphenyl)ethyl]-4*H*-1-benzopyran-4-one	—	33	—
108	*rel*-(5*R*,6*S*,7*R*)-5,6,7,8-Tetrahydro-5,6,7-trihydroxy-2-(2-phenylethyl)-4*H*-1-benzopyran-4-one	—	33	—
109	*rel*-(5*R*,6*S*,7*R*)-5,6,7,8-Tetrahydro-5,6,7-trihydroxy-2-[2-(4-methoxyphenyl)ethyl]-4*H*-1-benzopyran-4-one	—	33	—
	**Diepoxy-tetrahydro-2-(2-phenylethyl)chromones**			
110	5,6:7,8-Diepoxy-2-(2-phenylethyl)-5,6,7,8-tetrahydrochromone (oxidoagarochromone A)	72*, 79* and 81*	33	72*
111	5,6:7,8-Diepoxy-2-[2-(4-methoxyphenyl)ethyl]-5,6,7,8-tetrahydrochromone (oxidoagarochromone B)	72* and 79*	33	72*
112	5,6:7,8-Diepoxy-2-[2-(3-hydroxy-4-methoxyphenyl)ethyl]-5,6,7,8-tetrahydrochromone (oxidoagarochromone C)	72*	33	72*
113	5,6-Epoxy-7β-hydroxy-8β-methoxy-2-(2-phenylethyl)chromone	79*	—	—
114	(5*S*,6*R*,7*R*,8*R*)-2-(2-Phenylethyl)-7,8-epoxy-5,6-dihydroxy-5,6,7,8-tetrahydrochrome (tetrahydrochromone K)	81*	—	—
115	(5*R*,6*S*,7*S*,8*S*)-2-[2-(4′-Methoxyphenyl)ethyl]-7,8-epoxy-5,6-dihydroxy-5,6,7,8-tetrahydrochrome (tetrahydrochromone L)	81*	—	—
116	(5*R*,6*S*,7*S*,8*S*)-2-[2-(3′-Hydroxy-4′-methoxyphenyl)ethyl]-7,8-epoxy-5,6-dihydroxy-5,6,7,8-tetrahydrochrome (tetrahydrochromone M)	81*	—	—
117	5α,6α-Epoxy-7β,8α,3′-trihydroxy-4′-methoxy-2-(2-phenylethyl)chromone	83*	—	—
118	*rel*-(1*aR*,2*R*,3*R*,7*bS*)-1*a*,2,3,7*b*-Tetrahydro-2,3-dihydroxy-5-[2-(4-methoxyphenyl)ethyl]-7*H*-oxireno[*f*][1]benzopyran-7-one	84	33	—
119	*rel*-(1*aR*,2*R*,3*R*,7*bS*)-1*a*,2,3,7*b*-Tetrahydro-2,3-dihydroxy-5-(2-phenylethyl)-7*H*-oxireno[*f*][1]benzopyran-7-one	79*	33	—
120	Qinanmer	77	—	—
121	2-[2-(4-Glucosyloxy-3-methoxyphenyl)ethyl]chromone	78	—	—
122	(5*S*,6*S*,7*R*,8*S*)-2-(2-Phenylethyl)-6,7,8-trihydroxy-5,6,7,8-tetrahydro-5-[2-(2-phenylethyl)chromonyl-6-oxy]chromone (AH_10_)	—	92	—
123	(5*S*,6*S*,7*R*,8*S*)-2-(2-Phenylethyl)-6,7,8-trihydroxy-5,6,7,8-tetrahydro-5-[2-(2-phenylethyl)-7-hydroxy-chromonyl-6-oxy]chromone (AH_15_)	—	92	—
124	2,2′-Di-(2-phenylethyl)-8,6′-dihydroxy-5,5′-bichromone (AH_11_)	—	92	—
125	(5*S*,6*R*,7*R*,8*S*)-2-(2-Phenylethyl)-5,6,7-trihydroxy-5,6,7,8-tetrahydro-8-[2-(2-phenylethyl)-7-methoxychromonyl-6-oxy]chromone (AH_12_)	—	92	—
126	(5*S*,6*R*,7*R*,8*S*)-2-(2-Phenylethyl)-5,6,7-trihydroxy-5,6,7,8-tetrahydro-8-[2-(2-phenylethyl)chromonyl-6-oxy]chromone (AH_13_)	—	92	—
127	(5*S*,6*S*,7*S*,8*R*)-2-(2-Phenylethyl)-6,7,8-trihydroxy-5,6,7,8-tetrahydro-5-[2-(2-phenylethyl)-chromonyl-6-oxy]chromone (AH_14_)	—	92	—
128	Dioxin-linked bi-2-(2-phenylethyl)chromone (AH_21_)	—	97	—
129	Bi-(5*S*,6*S*,7*R*,8*S*)-2-(2-phenylethyl)-6,7,8-trihydroxy-5,6,7,8-tetrahydro-5-[2-(2-phenylethyl)chromonyl-6,7-dioxy]chromone (AH_18_)	—	93	—
130	AH19a	—	96	—
131	AH19b	—	96	—
132	AH20	—	95	—
133	(5*S*,6*R*,7*S*,8*R*)-2-[2-(4-Methoxyphenyl)ethyl]-5,6,7-trihydroxy-5,6,7,8-tetrahydro-8-{6-methoxy-2-[2-(3′′′-methoxy-4′′′-hydroxyphenyl)ethyl]chromonyl-7-oxy}chromone	82*	—	—
134	(5*S*,6*R*,7*S*,8*R*)-2-[2-(4-Methoxyphenyl)ethyl]-5,6,7-trihydroxy-5,6,7,8-tetrahydro-8-{2-[2-(4′′′-methoxyphenyl)ethyl]chromonyl-6-oxy}chromone	82*	—	—
135	(5*S*,6*R*,7*S*,8*R*)-2-(2-Phenylethyl)-5,6,7-trihydroxy-5,6,7,8-tetrahydro-8-[2-(2-phenylethyl)chromonyl-6-oxy]chromone	82*	—	—
136	(5*R*,6*R*,7*R*,8*S*)-2-(2-Phenylethyl)-5,6,7-trihydroxy-5,6,7,8-tetrahydro-8-[2-(2-phenylethyl)chromonyl-6-oxy]chromone	82*	—	—
137	Crassin A	—	—	100
138	(5*R*,6*S*,7*R*,8*S*)-Configuration (crassin B)	—	—	100
139	(5*S*,6*R*,7*S*,8*R*)-Configuration (crassin C)	—	—	100
140	Crassin D	—	—	100
141	Aquilacrassnin A	—	—	101
142	Aquilacrassnin B	—	—	101
143	Aquilacrassnin C	—	—	101
144	Aquilacrassnin D	—	—	101
145	Aquilacrassnin E	—	—	101
146	Aquilacrassnin F	—	—	101
147	(5*S*,6*R*,7*S*,8*R*)-(+)-Aquisinenone A	85	—	—
148	(5*R*,6*S*,7*R*,8*S*)-(−)-Aquisinenone A	85	—	—
149	(5*R*,6*S*,7*R*,8*S*)-(−)-4′-Methoxyaquisinenone A	85	—	—
150	(5*R*,6*S*,7*R*,8*S*)-(+)-Aquisinenones B	85	—	—
151	(5*S*,6*R*,7*S*,8*R*)-(−)-Aquisinenones B	85	—	—
152	(5*S*,6*R*,7*S*,8*R*)-(−)-6′′-Hydroxyaquisinenone B	85	—	—
153	(5*R*,6*S*,7*R*,8*S*)-(+)-6′′-Hydroxy-4′,4′′′-dimethoxyaquisinenone B	85	—	—
154	(5*R*,6*S*,7*R*,8*S*)-(+)-Aquisinenones C	85	—	—
155	(5*S*,6*R*,7*S*,8*R*)-(−)-Aquisinenones C	85	—	—
156	(5*S*,6*R*,7*S*,8*R*)-(−)-Aquisinenone D	85	—	—
157	(5*R*,6*S*,7*R*,8*S*)-4′-Demethoxyaquisinenone D	85	—	—
158	(5*S*,6*R*,7*S*,8*R*)-4′-Demethoxyaquisinenone D	85	—	—
159	(5*S*,6*R*,7*S*,8*R*)-(+)-Aquisinenone E	85	—	—
160	(5*S*,6*R*,7*S*,8*R*)-(−)-Aquisinenone F	85	—	—
161	(5*S*,6*R*,7*S*,8*R*)-(−)-Aquisinenone G	85	—	—
162	(+)-4′-Methoxyaquisinenone G	85	—	—

a *A. s*, *A. m*, and *A. c* indicate *A. sinensis*, *A. malaccensis*, and *A. crassna*, respectively.

b The reference was not found.

c “*” indicates that the agarwood in this article was artificial agarwood.

Regarding the study of chromones, most researchers use agarwood extracts, usually ethanol (EtOH) extracts, to extract and separate the monomers. The structures of the compounds are determined by a series of assays, including LC/MS, and nuclear magnetic resonance.

### 3.1. Chromones in *A. sinensis*

Approximately 130 chromone derivatives have been obtained from *A. sinensis*, comprising 22 forms of 2-(2-phenylethyl)chromones, six 5,6,7,8-tetrahydro-2-(2-phenylethyl)chromones, and three diepoxy-tetrahydro-2-(2-phenylethyl)chromones.

Yang *et al.* obtained 1–6 from an EtOH extract of *A. sinensis*, which belong to the group of 2-(2-phenylethyl)chromones;^65^ they later extracted 7–9 from an EtOAc–EtOH extract.^66^ In addition, 10–12 were isolated by Liu *et al.*^67,68^ Dai *et al.* extracted 165–168 from the same species.^69–71^ Yagura and coworkers obtained four chromones, 13, 14, 70 and 71, in 2003 and later extracted 110–112;^72^ these are all diepoxy tetrahydrochromones. In 2012, Yang and coworkers isolated eight new chromone derivatives, 15–22.^73^ Gao *et al.*^74^ and Chen *et al.*^75^ isolated 59, 69, and aquilarones A–I (64–66, 74, 98–101, 106), with two known chromones, 15 and 21, from an EtOH extract of resinous wood of *A. sinensis*. Yang^76^ obtained 2-(2-phenylethyl)chromone derivatives 1, 2, 4, 13, 23, 22, and 36–42 from a Et_2_O extract of “Qi-Nan”. Later, this research team^77,78^ found a new compound, 120, comprising 2-(2-phenylethyl)chromone and sesquiterpene moieties, named “Qinanmer”; a 2-(2-phenylethyl)chromone glycoside, 121, together with two 2-(2-phenylethyl)chromone derivatives, 77 and 79, were obtained from a EtOH extract of “Qi-Nan”.

Since 2014, researchers have been engaged in the study of artificial agarwood induced by the holing method. Li *et al.*^79^ isolated three previously undescribed 2-(2-phenylethyl)chromone derivatives, 30, 31, and 113, and thirteen 5,6,7,8-tetrahydro-2-(2-phenylethyl)chromones, named tetrahydrochromones A–M (86–95, 114–116), together with thirteen known ones (2, 4–6, 10, 24, 28, 32, 33, 110, 111, 118, and 119) from an EtOAC extract. Liao *et al.*^80,81^ used the same method and found 2-(2-phenylethyl)chromone derivatives 12, 15, 16, 19, 42, 47–53, 61–63, 77, 81, 96–101, and 110. The EtOAc fraction also contained four new bi-phenylethylchromones, 133–136.^82^ Kuang *et al.*^48^ were also interested in agarwood induced by artificial holing; they researched the chemical constituents of the *n*-butanol fraction of an EtOH extract and obtained 9, 74, and 82.

Liu *et al.*^83^ separated and identified 44–46 and 117 from an EtOH extract of agarwood produced *via* the whole-tree agarwood-inducing technique.

Huo and coworkers^84^ isolated 2-(2-phenylethyl)chromone derivatives 1, 7, 23, 43, 58–60, 71, 76, 99, 100, 102–105, and 118 from a 95% EtOH–EtOAc extract of resinous wood of *A. sinensis*. Subsequently, through LC-MS-guided separation and purification, they obtained sixteen new 2-(2-phenylethyl)chromone dimers, including four pairs of enantiomers, along with eight optically pure analogues (151–162).^85^ Wang *et al.*^51^ isolated compounds 54–57, which belong to the group of 2-(2-phenylethyl)chromone derivatives, from resinous wood, together with five known compounds, 1, 6, 9, 29, and 58, from a MeOH extract.

### *3.2*. Chromones in *A. malaccensis (or A. agallocha)*

More than 30 chromones have been reported from *A. agallocha*, of which nine are the same as in *A. sinensis*, namely 1,^63^ 2, 3,^86^ 5, 6,^86^ 9,^87^ and 71 and 72.^87^ Since 1982, Shimada and coworkers have been engaged in the isolation of chromones 2–3, 5–6, 77–78,^88^ 82,^89^ and 83.^89^ In 1986, Nakanishi isolated a known chromone, 23, and a new chromone, 24.^90^ Then, 7, 9, 85,^91^ 122–127,^92^ and 129 (ref. 93) were isolated and identified. Konishi devoted himself to this work, also aiding other researchers in the field; from 1989 to 1992, he found 79,^94^ 80, 81, 132,^95^ 130–131,^96^ 128,^97^ and 71–72.^87^ Iwagoe obtained 123 and 129,^93^ and in 2005, Alkhathlan isolated 3, 6, and 25 from *A. agallocha*.^98^

The chromones isolated from *A. malaccensis* were mainly reported by T. Konishi in 2002, namely 1, 26–29, and 10.^99^ Wu *et al.*^33,50^ reported the 2-(2-phenylethyl)-4*H*-chromone derivatives 1–3, 5, 6, 9, 14, 23, 24, 58, 67, 68, 97, 107–112, 118, and 119 from a 70% MeOH extract of *A. malaccensis* agarwood.

### 3.3. Chromones in *A. crassna*

There are few reports on chromones in *A. crassna*. Diepoxy-tetrahydro-2-(2-phenylethyl) chromones 110–112 were obtained from *A. crassna*.^72^ Yang *et al.*^100,101^ obtained four new bi-2-(2-phenylethyl)chromone derivatives, crassins A–D (137–140), and six previously undescribed uncommon ester-bonded dimeric compound aquilacrassnins A–F (141–146) from the EtOAc extract of agarwood originating from *A. crassna.*

## 4. Discussion

**Fig. 1:**
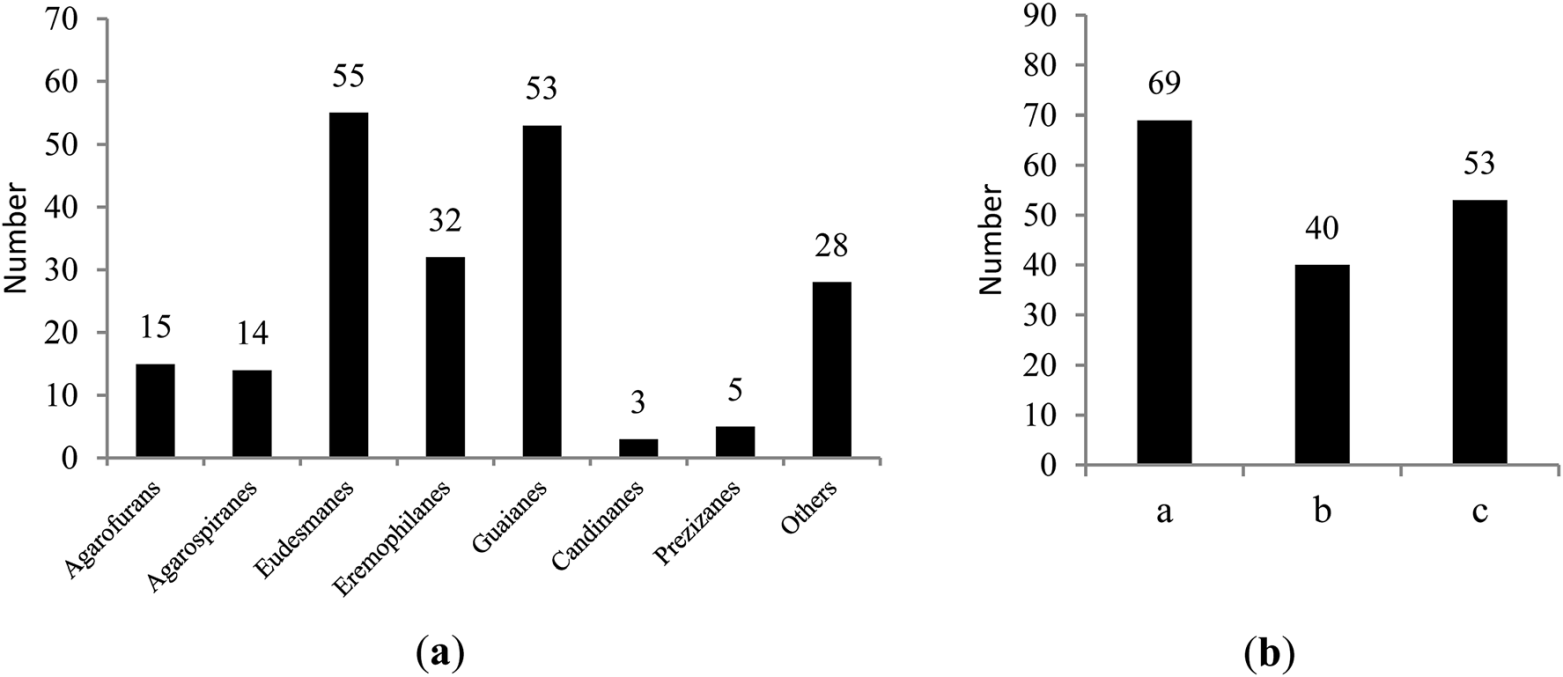
(a) The number of different types of sesquiterpenes in agarwood; (b) the number of different types of chromone derivatives in agarwood ((a) 2-(2-phenylethyl)chromones; (b) 5,6,7,8-tetrahydro-2-(2-phenylethyl)chromones; (c) others).

Among the 367 new main chemical constituents from agarwoods that were statistically assessed in this paper, chromone derivatives and sesquiterpenes accounted for 44.14% and 55.86%, respectively, of the total constituents. It can be seen in Fig. 1(a) that the largest numbers of sesquiterpenes in agarwood are eudesmanes, guaianes and eremophilanes. Fig. 1(b) reflects the number of different chromones in agarwood, where 2-(2-phenylethyl)chromones are currently the most commonly isolated types.

**Fig. 2:**
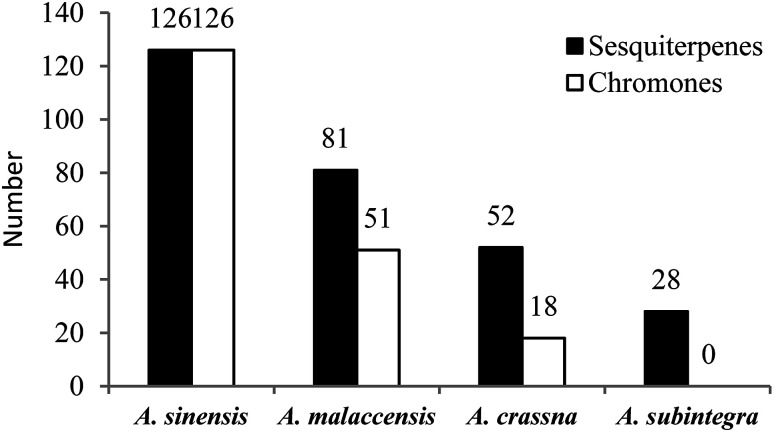
The numbers of sesquiterpenes and chromone derivatives in different species of agarwood.

From the statistical results (shown in Fig. 2), researchers are currently mainly engaged in the study of the chemical constituents of agarwood originating from *A. sinensis*, *A. malaccensis*, and *A. crassna*, respectively, of which most of the new compounds were isolated from *A. sinensis*. It can be seen that resources are important prerequisites for the study of agarwood; thus, there are many studies on species with relatively abundant resources, such as *A. sinensis*, *A. malaccensis*, and *A. crassna*. Of course, this is also closely related to geographical distribution. Agarwoods originating from different *Aquilaria* plants contain some common compounds as well as some different compounds. Among different species of agarwood, the chemical compositions are quite different. Therefore, it is necessary to indicate the species from which the used agarwood is derived. However, during the writing process, we found that many articles on the separation of compounds from agarwood did not indicate which species of the genus *Aquilaria* the agarwood was derived from. Therefore, we encourage researchers studying agarwood to indicate more information about the origin and tree species to clarify the source of the material.

According to the data, the number of sesquiterpenes isolated from agarwood has thus far been higher than the number of chromones, and the proportion of articles is also the same. In the past 10 years, the number of articles on chromones has increased rapidly. The study of the chemical constituents of agarwood from sesquiterpenes to chromone derivatives shows that increasing numbers of researchers are beginning to focus on revealing the main components of agaric pharmacologically active substances rather than only fragrance components. Therefore, we can see that research on the separation and activity of chromone derivatives still has broad research prospects.

By summarizing and comparing the chemical compositions of different tree species, we can provide more research ideas. The same components can be used as standards for quality assessment, with reliable and stable characteristics, and different components can guide the selection of high quality agarwood species. By reviewing the chemical compositions of agarwoods from the four species, we believe that the following characteristics should be considered when selecting quality control standards. Due to the complex composition of agarwood, sesquiterpenes and chromone derivatives should be considered first, especially chromones, mainly because they are characteristic components of agarwood, and chromone derivatives are easier to separate and preserve. Due to the extremely complex sources and types of agarwood, researchers in different countries should fully consider the common chemical composition when selecting control indicators and formulating quality testing methods to improve the scope and scientificity of the testing methods, such as F2 and F3. Of course, even with the limited amounts of research on individual species, it is possible to flexibly select components, such as chromone 1.

## 5. Conclusion

Agarwood, which is expensive and widely used, is derived from the resin-containing wood of *Aquilaria* species trees. The chemical components of agarwood are diverse and complex; 367 new chemical constituents from agarwood were statistically assessed in this paper. This review summarizes the main molecular skeletons of agarwood compounds, revealing the differences in the chemical compositions of agarwood originating from different *Aquilaria* species. This will help researchers to better understand research on agarwood and select more suitable detection indicators.

With the continuous exploration and efforts made by scientists in recent years, the understanding of the chemical compositions of agarwood from different sources is continuously improving, and some specific chemical compositions may become identification indices and judgement standards of agarwood samples from different sources. In the future, we expect to see more research on the chemical components of agarwood from different species in order to help identify characteristic compounds of agarwood, establish a stable, effective, comprehensive, and reliable quality evaluation system, and consequently elucidate which species best produce agarwood.

## Conflicts of interest

The authors declare no conflict of interest.

## Supplementary Material
